# Comparative Study on Photoinhibition Characteristics and Adaptation Mechanisms of New Current-Year Needles of *Pinus densiflora* var. *zhangwuensis* and *Pinus sylvestris* var. *mongolica* in Sandy Land Environments

**DOI:** 10.3390/plants15192953

**Published:** 2026-09-28

**Authors:** Peng Meng, Mengyu Zhang, Xiaoyun Wang, Jing Liu

**Affiliations:** 1 School of Landscape Architecture and Forestry, Qingdao Agricultural University, Qingdao 266109, China; mengpeng18@163.com (P.M.); 18764462331@163.com (M.Z.); wxy5127@163.com (X.W.); 2 Shandong Key Laboratory for Germplasm Innovation of Saline-Alkaline Tolerant Grasses and Trees, Qingdao 266109, China; 3 College of Civil Engineering & Architecture, Qingdao Agricultural University, Qingdao 266109, China

**Keywords:** chlorophyll a fluorescence, daily change, photoinhibition, *Pinus densiflora* var. *zhangwuensis*, *P. sylvestris* var. *mongolica*, tracheid morphology

## Abstract

Whether photoinhibition occurs in tall trees of *Pinus densiflora* var. *zhangwuensis* and *P. sylvestris* var. *mongolica* in summer and their response mechanisms to strong midday light in sandy land are not yet clear. Therefore, three standard trees were selected for each species as biological replicates by randomly setting up standard plots. Daily changes in the chlorophyll a fluorescence parameter of current-year needles (CYNs) and one-year needles (OYNs) in different parts of the standard tree crowns (in sun branches and shade branches) of the two species were comparatively studied, and the potential hydraulic transport capacities of the sample branches were also evaluated. The results are as follows: (1) The daily changes in the maximal photochemical efficiency of PSII (F_v_/F_m_) in the sun CYNs of *P. sylvestris* var. *mongolica* obviously presented a descending–ascending trend, and its lowest value was observed at noon. The maximum non-photochemical quenching (NPQ) of the sun CYNs in this species was observed at noon, and an NPQ of greater than 1 lasted for 4–5 h. (2) The midday F_v_/F_m_ of the sun CYNs in *P. densiflora* var. *zhangwuensis* was close to the morning and shade CYN levels. Compared with *P. sylvestris* var. *mongolica*, the NPQ of the sun CYNs of *P. densiflora* var. *zhangwuensis* remained low under the condition of high light at noon, but the number of active reaction centers per unit cross-sectional area (RC/CS_m_) and the electron transfer efficiency (F_m_/F_o_) were higher. Further analysis of the variation in the light energy absorbed by each reaction center (ABS/RC) showed that the variation in the NPQ in the sun CYNs of the two species was very similar to that of ABS/RC, and the two indices had a very significant positive correlation (the correlation coefficients of *P. sylvestris* var. *mongolica* and *P. densiflora* var. *zhangwuensis* were 0.7535 and 0.7193, respectively, *p* < 0.001). (3) Through the comparison of the tracheid morphologies, our study also found that the outer and inner diameters of the pit of *P. densiflora* var. *zhangwuensis* were 1.26 and 1.28 times those of the pit of *P. sylvestris* var. *mongolica*, respectively, and the theoretical hydraulic conductance (Kt) in the sun branches of the former was 6.32 times that of the latter. The above results suggested that photoinhibition might also be involved in the tall trees of *P. sylvestris* var. *mongolica*, while *P. densiflora* var. *zhangwuensis* had a more effective mechanism to avoid photoinhibition.

## 1. Introduction

*Pinus sylvestris* var. *mongolica* is one of the main coniferous trees introduced to and cultivated in the sandy land of the ‘three north’ regions of China. The planting area is large, but it has declined in some mature forests, and its decline mechanism is not very clear. *P. densiflora* var. *zhangwuensis* is a new natural hybrid variety of *P. densiflora* [1]. It is a national forest variety of China and has the characteristics of fast growth and no tree decline when compared with *P. sylvestris* var. *mongolica* of the same age. Therefore, it has been introduced to and cultivated in the sandy lands of many provinces and autonomous regions in China, including Tibet, and has been extended to other countries, including Pakistan. With the increasing market demand for *P. densiflora* var. *zhangwuensis*, the number of studies comparing its physiological and ecological characteristics with those of *P. sylvestris* var. *mongolica* is also increasing, and the mechanisms of its stress resistance and fast growth have been gradually revealed. In terms of stress resistance, the authors of this paper carried out a comparative study on the drought resistance, cold resistance, and saline–alkali resistance of the two tree species and found that *P. densiflora* var. *zhangwuensis* exhibited stronger resistance [2]. To assess the mechanism behind the fast growth of these species, one of our previous studies used an Li-6400 photosynthetic analyzer to compare the photosynthetic characteristics of the two species and confirmed that, compared with the same age of *P. sylvestris* var. *mongolica* of the same age, an 18-year-old *P. densiflora* var. *zhangwuensis* tree exhibits stronger photosynthesis and a higher light saturation point (LSP) in sandy summer environments [3]. High light, high temperature, and low humidity are the main characteristics of sandy land in summer, and many plants exhibit photoinhibition under high light intensity [4]. Therefore, we propose the hypothesis that there may be no photoinhibition in tall trees of *P. densiflora* var. *zhangwuensis* in summer sandy environments because its LSP is higher, whereas *P. sylvestris* var. *mongolica* may have photoinhibition. One of the aims of this paper is to verify this hypothesis.

In the past, the definition of photoinhibition has referred to the phenomenon that the photosynthetic rate of plants decreases at noon under high light intensity [5], and it has been found that many plants demonstrate the photoinhibition phenomenon according to this definition, which is also equivalent to the phenomenon of the midday depression of photosynthesis. However, there are many reasons for the occurrence of the midday depression of photosynthesis, including stomatal closure limiting CO_2_ supply, strong light-induced photoinhibition, high-temperature-inhibited enzyme activity, and increased water stress. These factors are intertwined, and it is difficult to judge whether the midday depression of photosynthesis is simply caused by high light intensity [6].

To date, photosystem II (PSII) has been recognized as the part most vulnerable to photoinhibition [7]. With the emergence of chlorophyll a fluorescence analysis technology, people can monitor the function of PSII in real time and non-destructively, thereby facilitating a deeper understanding of photoinhibition. Under high light conditions, the quinone receptors in PSII are largely reduced, resulting in a decrease in primary photochemical efficiency. The rate of light absorption exceeds the ability of photosynthesis, which in turn leads to photooxidative damage to PSII. This process is called photoinhibition. The maximum photochemical efficiency (F_v_/F_m_) index measured by the chlorophyll a fluorescence technique is often used to indicate photoinhibition because this index is not affected by the photorespiration rate [8]. Some chlorophyll a fluorescence indices such as F_v_/F_m_ are more reflective of ‘intrinsic’ characteristics than ‘apparent’ gas exchange indices [9]; thus, they are often applied to decipher photosynthetic and adaptation mechanisms in the field of photoinhibition research [10,11].

Three main adaptation mechanisms of plants that avoid photoinhibition or alleviate its damage include the repair of key proteins in the reaction center (RC) [12], heat dissipation [13], and light energy transfer [14]. The important fluorescence indices involved in this field include F_v_/F_m_, the number of active RCs per unit leaf cross-section (RC/CS_m_), the light energy absorbed by each RC (ABS/RC), non-photochemical quenching (NPQ), and electron transfer efficiency (F_m_/F_o_) [15]. These indices are helpful to deeply understand how plants avoid the occurrence of photoinhibition, or how they deal with excess light energy in the process of photoinhibition to reduce the damage to their own PSII. When the photosynthetically active radiation (PAR) is absorbed by the light-harvesting complex (LHC), the absorbed energy is transferred to the RCs of photosystem I (PSI) and PSII in the form of excitons [16]. The primary damage from photoinhibition occurs within the RCs of PSII [17]. Under high light intensity, plants with a larger RC/CS_m_ value have higher photosynthetic apparatus integrity, which is conducive to utilizing light energy and avoiding the occurrence of photoinhibition. When photoinhibition occurs in plants, due to the blocking of photochemical processes, excess light energy is released into the external space in the form of thermal energy. This process is called NPQ. This mechanism exists not only in plants but also in some algae and bacteria [18]. Therefore, it is an ancient mechanism used by organisms to adapt to high light intensity. Recent studies have shown that the process of NPQ in some plants can be stopped quickly when they are transferred to normal light intensity, which can ensure strong photosynthesis [19]. In the F_m_/F_o_ index, F_o_ stands for the chlorophyll a fluorescence level with oxidized QA, and F_m_ stands for the fluorescence level with completely reduced QA. F_m_/F_o_ can reflect the overflow level of the excitation energy in the PSII antenna system to the PSI antenna system. The larger the value, the lower the overflow level [9], that is, the higher the electron transfer efficiency. In addition to the above three adaptation mechanisms, studies have shown that leaves are often prone to photoinhibition when they are in a water-deficient state [20]. Therefore, the potential hydraulic transport capacity of plants is also one of the important adaptation mechanisms. The structural analysis of the conducting tissue (such as tracheids of conifers) in leaves and adjacent branches under high light intensity assesses the potential hydraulic transport capacity of plants [21].

For coniferous trees, sun (shade) needles refer to needles that grow in a strong (weak) light environment for a long time and adapt to this environment, usually growing in the upper layer (inside or at the bottom) of the canopy [22]. The newborn needles are more sensitive to photoinhibition; however, there are relatively few studies on newborn needles. As mentioned above, chlorophyll a fluorescence technology is suitable for evaluating plant high-light adaptability, and it can monitor the same leaf (sun or shade leaves) for a long time. However, this technology is mostly applied to the study of seedling or young tree stages, while there are few studies focusing on large trees due to the limitation of tree height.

To sum up, in this study, the daily variations in chlorophyll a fluorescence parameters of sun and shade current-year needles (CYNs) and one-year needles (OYNs) of *P. densiflora* var. *zhangwuensis* and *P. sylvestris* var. *mongolica* were dynamically monitored, and the characteristics of their branch tracheids were also compared to answer the following two questions: 1. Whether the two species exhibit photoinhibition under high light intensity in sandy land in summer? 2. Whether their adaptation mechanisms in response to photoinhibition are consistent?

## 2. Results and Analysis

### 2.1. Daily Changes in F_v_/F_m_

The daily variation in the F_v_/F_m_ value of sun CYNs of *P. sylvestris* var. *mongolica* was large, and the maximum amplitude (the ratio of daily variation to maximum value) was 28.99 ± 6.28%. The overall change trend showed a single-valley curve, and the trough appeared in the range of 11:00–13:00, in which the F_v_/F_m_ level was significantly lower than at other times. The lowest value of F_v_/F_m_ appeared at 12:00 and was significantly lower than that at 11:00 and 13:00, indicating that the F_v_/F_m_ value of *P. sylvestris* var. *mongolica* decreased more significantly at 12:00 with the strongest light intensity (Figure 1A). After one year, the diurnal variation in the F_v_/F_m_ value of sun OYNs increased further, and the maximum amplitude reached 30.13 ± 1.44%. Overall, it was still a single-valley curve, but the time range of the trough was delayed, in the interval of 13:00–16:00. The lowest F_v_/F_m_ value appeared at 15:00, and the value was lower than the lowest value of the CYNs. F_v_/F_m_ recovered after 15:00, but until 17:00, it was still significantly lower than that in the morning (Figure 1C).

The daily variation in the F_v_/F_m_ value of sun CYNs of *P. densiflora* var. *zhangwuensis* was relatively small, with a maximum amplitude of 16.88 ± 2.70%, and the change trend showed a double-valley curve, with two relatively small troughs at 10:00 and 14:00, respectively. At 11:00–13:00, with strong light intensity, the F_v_/F_m_ value of sun CYNs of *P. densiflora* var. *zhangwuensis* was high, without any significant difference from the value at 8:00 or 9:00 (Figure 1A). The daily variation range of F_v_/F_m_ of sun OYNs of *P. densiflora* var. *zhangwuensis* was further narrowed, with the maximum amplitude of 7.91 ± 4.10%, and the mean value of each moment exceeded 0.70 (Figure 1C).

The F_v_/F_m_ values of shade CYNs of the two species remained high, that is, above 0.75, and the daily variations were smaller, with the maximum amplitude of 7.07 ± 4.60% (*P. densiflora* var. *zhangwuensis*) and 5.11 ± 0.92% (*P. sylvestris* var. *mongolica*), respectively. The F_v_/F_m_ values between the two species were not significantly different (except at 13:00), and the value for *P. densiflora* var. *zhangwuensis* was significantly higher than that of *P. sylvestris* var. *mongolica* at 13:00 (Figure 1B). The daily variation range of F_v_/F_m_ of shade OYNs of the two species was further narrowed, with the maximum amplitude of 6.49 ± 1.52% (*P. densiflora* var. *zhangwuensis*) and 3.27 ± 0.88% (*P. sylvestris* var. *mongolica*), respectively, and there was no significant difference between the two species at each time point (Figure 1D).

The above analysis showed that, when the light intensity was high at noon, no photoinhibition occurred in the shade CYNs of the two species, and no photoinhibition occurred in the sun CYNs of *P. densiflora* var. *zhangwuensis*. Contrarily, the sun CYNs of *P. sylvestris* var. *mongolica* showed obvious photoinhibition. Thus, we endeavor to answer the following two questions: What is the mechanism used by the sun CYNs of *P. densiflora* var. *zhangwuensis* to avoid photoinhibition? What is the mechanism used by the sun CYNs of *P. sylvestris* var. *mongolica* to alleviate photoinhibition damage?

### 2.2. The Exploration of Photoinhibition Characteristics and Adaptation Mechanisms

#### 2.2.1. Daily Changes in ABS/RC and RC/CS_m_

The ABS/RC values of the sun CYNs of the two species were significantly greater than those of the shade CYNs at most time points (Table 1). The average ABS/RC of the sun CYNs of *P. sylvestris* var. *mongolica* was 2.02 ± 0.26, and the ratio of the average ABS/RC of the sun CYNs to that of the shade CYNs was 1.22 ± 0.15, while that of *P. densiflora* var. *zhangwuensis* was only 1.13 ± 0.11. The average ABS/RC in the sun CYNs of *P. sylvestris* var. *mongolica* was slightly larger than that of *P. densiflora* var. *zhangwuensis* (1.98 ± 0.23), especially at noon (e.g., 11:00 and 13:00), and the ABS/RC values of the sun CYNs of the former were significantly larger than those of the latter (*p* < 0.05).

At most time points, the RC/CS_m_ values of the sun CYNs of the two species were significantly lower than those of the shade CYNs (*p* < 0.05, Table 1). At noon under high light intensity (11:00 and 13:00), this index of the sun CYNs of *P. densiflora* var. *zhangwuensis* remained high, close to that of shade CYNs, which was a strategy that enabled its full and effective use of light energy. Under high light intensity, its two strong fluctuations also indicated that its photosynthetic apparatus integrity can be quickly restored to the level of 8:00 at 17:00 (Table 1). In fact, the RC/CS_m_ values in both sun and shade CYNs of *P. densiflora* var. *zhangwuensis* were higher than those of *P. sylvestris* var. *mongolica*, and the latter had the lowest RC/CS_m_ in sun CYNs under high light intensity, and it did not return to the level of 8:00 at 17:00 (Table 1).

#### 2.2.2. Daily Changes in NPQ and the Correlation Between NPQ and ABS/RC

As shown in Figure 2A, the daily mean value of NPQ in the sun CYNs of *P. sylvestris* var. *mongolica* was 1.389 ± 1.121, and the daily change showed a single peak curve that increased first and then decreased. The peak appeared at 12:00 when the light intensity was the highest, which was 3.741 ± 1.56. The time range of an NPQ value greater than 1 was up to 4–5 h, and it was significantly higher than that of *P. densiflora* var. *zhangwuensis* in the range of 11:00–13:00. The daily mean value of NPQ of the sun CYNs of *P. densiflora* var. *zhangwuensis* was 0.787 ± 0.738, and the daily change showed a fluctuating upward trend. Only at two time points did its NPQ exceed 1: the first time point was 10:00 (1.034 ± 0.170), and then its NPQ quickly decreased; the second time point was 15:00 (2.722 ± 0.434), and then its NPQ decreased quickly.

The NPQ values of shade CYNs of the two species remained low (significantly lower than those of the sun CYNs most of the time), showing fluctuating changes, but both peaked at 15:00, and the peak values were lower than 2.5. The daily mean values of NPQ in the shade CYNs of *P. sylvestris* var. *mongolica* and *P. densiflora* var. *zhangwuensis* were 0.769 ± 0.563 and 0.554 ± 0.422, respectively. At 13:00, the NPQ value of *P. densiflora* var. *zhangwuensis* was as low as 0.187 ± 0.157, which was significantly lower than that of *P. sylvestris* var. *mongolica*. At other time points, there was no significant difference in NPQ values between the two species (Figure 2B).

In sun needles, the NPQ values of the two species were very significantly positively correlated with ABS/RC (*p* < 0.001; Figure 3A), and the correlation coefficient (r) was 0.8015 (*P. densiflora* var. *zhangwuensis*) and 0.7677 (*P. sylvestris* var. *mongolica*). In the shade needles, the correlation between the two indices of the two species decreased, but the two indices in *P. densiflora* var. *zhangwuensis* were still significantly positively correlated (*p* < 0.05, Figure 3B). Whether in sun needles or shade needles, the fitting degree (R^2^) of *P. densiflora* var. *zhangwuensis* was higher than that of *P. sylvestris* var. *mongolica*. Further general linear model univariate analysis showed that ABS/RC had a significant effect on NPQ in both sun and shade needles, and for this effect, there was a significant difference between the two species, indicating that the NPQ of *P. densiflora* var. *zhangwuensis* needles was more sensitive to ABS/RC (Table 2).

#### 2.2.3. Daily Changes in F_m_/F_o_

As shown in Figure 4A, F_m_/F_o_ of the sun CYNs of *P. densiflora* var. *zhangwuensis* decreased with the increase in light intensity and reached its lowest value at about 10:00. Although the light intensity was further enhanced, the values of this index increased rapidly and returned to the level close to that in the morning at 11:00-13:00. Then, the index decreased and later returned to its level in the morning at 17:00. The F_m_/F_o_ of the sun CYNs of *P. sylvestris* var. *mongolica* decreased with the increase in light intensity, reaching its lowest value at about 12:00, and then gradually increased due to the decrease in light intensity, but did not fully recover to its level in the morning.

Compared with the sun CYNs, the daily variation in F_m_/F_o_ of the shade CYNs of the two species was significantly smaller, and the lowest values of both sun and shade CYNs appeared at about 15:00. The F_m_/F_o_ values of *P. densiflora* var. *zhangwuensis* were significantly higher than those of *P. sylvestris* var. *mongolica* at 11:00–13:00 in both sun and shade CYNs, especially in sun CYNs, and the average F_m_/F_o_ value of the former was 1.56 times that of the latter (Figure 4A,B).

#### 2.2.4. Comparison of Tracheid Morphology and Evaluation of Potential Hydraulic Transport Capacity of Branches Between the Two Species

Except for tracheid length, there was no significant difference in other tracheid morphological indices of shade branches between the two species. In sun branches, the tracheid length, width, and lumen diameter of *P. densiflora* var. *zhangwuensis* were significantly higher than those of *P. sylvestris* var. *mongolica*, that is, 1.55, 1.44, and 1.58 times, respectively (Table 3). From the point of view of the morphology and distribution of pits, both species have bordered pits and a typical pinoid pit distribution (Figure 5). The outer and inner diameters of the pits in the sun branches of *P. densiflora* var. *zhangwuensis* were significantly higher than those of *P. sylvestris* var. *mongolica*, that is, 1.26 and 1.28 times. It is worth noting that the calculated Kt value of *P. densiflora* var. *zhangwuensis* was 6.32 times that of *P. sylvestris* var. *mongolica* (Table 3). Based on the above indicators, it can be inferred that the potential hydraulic transport capacity of the sun branches of *P. densiflora* var. *zhangwuensis* is very high.

## 3. Discussion

### 3.1. Photoinhibition in P. sylvestris var. mongolica in Summer

By monitoring the daily variations in chlorophyll a fluorescence indices, information regarding the response of the plant photosynthetic apparatus to environmental factors such as light and temperature can be obtained [23]. This study found that the F_v_/F_m_ of sun CYNs of *P. sylvestris* var. *mongolica* decreased significantly at noon (Figure 1A), indicating that its photosynthesis was inhibited by light, and the degree of photoinhibition increased with the increase in light intensity. The main reason for this phenomenon is the reversible inactivation of PSII and the decrease in the number of active RCs at noon (Table 1), resulting in a decrease in the efficiency of light energy conversion. The NPQ of *P. sylvestris* var. *mongolica* was higher (especially at noon), lasted for a long time, and did not stop after the light intensity decreased, which meant that the species also protected PSII through a heat dissipation mechanism to reduce its damage. In addition, the F_m_/F_o_ value of *P. sylvestris* var. *mongolica* is lower, indicating that the excitation energy in its PSII antenna system has a higher level of spillover to the PSI antenna system, which is another mechanism for its adaptation to high light intensity. The above two mechanisms work together in *P. sylvestris* var. *mongolica*. Although they do not avoid photoinhibition, they reduce the degree of damage to PSII. The F_v_/F_m_ and RC/CS_m_ values of this species gradually increased in the afternoon, indicating that the damage to its PSII in CYNs was reversible under the natural conditions of sandy land without soil water stress. However, the above two protection mechanisms also make *P. sylvestris* var. *mongolica* lose more light energy, which inevitably reduces its photosynthetic capacity and its growth (Table 4). The F_v_/F_m_ value of the sun OYNs of *P. sylvestris* var. *mongolica* showed that the phenomenon of photoinhibition still existed; the occurrence time was delayed, but the degree of damage to the photosystem was further increased, which manifested as a decrease in the minimum value and an increase in the recovery time (Figure 1C). It has been reported that the growth of *P. sylvestris* var. *mongolica* was reduced and its life cycle was shortened after it was introduced and planted in sandy land, and there was also a decline in the natural forest of sandy land where water stress rarely occurs [24,25]. This should be partly related to photoinhibition because the sandy land environment has sunny weather, coupled with the sunlight reflection of white sand, and is often able to reach a PAR range of 1500–2500 μmol·m^−2^·s^−1^ (Table 5).

### 3.2. Possible Adaptation Mechanism of P. densiflora var. zhangwuensis to Strong Light Intensity

Unlike *P. sylvestris* var. *mongolica*, the midday F_v_/F_m_ of sun CYNs of *P. densiflora* var. *zhangwuensis* was larger, indicating that it was almost not subject to photoinhibition. When the light intensity reached 2000 μmol·m^−2^·s^−1^ at noon, the number of active RCs in this species was high, which was an important response mechanism to strong light irradiation, and this mechanism has also been reported in other sandy plants [26]. Based on this study, this mechanism is also the most effective light protection pathway for *P. densiflora* var. *zhangwuensis* because it basically eliminates the occurrence of photoinhibition, which is consistent with previous reports [12]. The fast repair ability of inactivated RCs of *P. densiflora* var. *zhangwuensis* may be due to its higher antioxidant capacity [27], and our previous studies have found high catalase activity in the needles of this species [2]. The mean value of NPQ in the sun CYNs of *P. densiflora* var. *zhangwuensis* was small, especially at noon, which was significantly lower than that of *P. sylvestris* var. *mongolica* (Figure 2). The sun CYNs of *P. densiflora* var. *zhangwuensis* maintained a low level of NPQ, indicating that its heat dissipation consumed little energy, thus ensuring that the photosynthetic rate was maintained at a high level, and our previous studies have proved that the daily photosynthetic yield of the sun needles of *P. densiflora* var. *zhangwuensis* in summer is 2.1 times that of *P. sylvestris* var. *mongolica* [3], which was also an important reason why its height growth and diameter growth were significantly higher than those of *P. sylvestris* var. *mongolica* (Table 4). Our study also found that the NPQ of the two species was not linearly positively correlated with light intensity but with ABS/RC (Figure 3), indicating that the initiation mechanism of NPQ may have emerged from the interior of PSII, as stated in other studies [28,29]. Due to the high number of active RCs in the sun CYNs of *P. densiflora* var. *zhangwuensis*, the maximum ABC/RC did not appear at noon but appeared in the afternoon, which led to a lag in NPQ initiation, and its peak NPQ also appeared at about 15:00 (Figure 2A and Table 1). The positive correlation between the two indices of *P. densiflora* var. *zhangwuensis* was significantly higher than that of *P. sylvestris* var. *mongolica*, indicating that the NPQ regulation mechanism of this species based on the number of active RCs was more sensitive, which made its NPQ not remain high for a long time. Its NPQ peak in the afternoon may also be partially caused by high temperature (Table 5). Studies have shown that high temperature can reduce the number of active RCs [30,31]. The electron transfer efficiency value (F_m_/F_o_) of *P. densiflora* var. *zhangwuensis* is high (Figure 4), and the spillover level of excitation energy from the PSII antenna system to the PSI antenna system is low, which also partially contributes to its higher photosynthetic capacity.

The growth indices of tracheids and pits of the sun branches of *P. densiflora* var. *zhangwuensis* were higher (Figure 5), and its theoretical hydraulic conductance (Kt) was 6.32 times that of *P. sylvestris* var. *mongolica*, indicating that it had greater potential hydraulic transport capacity (Table 3). Studies have shown that greater potential hydraulic transport capacity is an effective mechanism used by conifers to endure drought stress, and it is often positively correlated with photosynthetic capacity [32,33]. In addition, our previous reports have also found that *P. densiflora* var. *zhangwuensis* is characterized by deep roots [34], which also helps it avoid drought stress. The above strategies can make the sun CYNs of *P. densiflora* var. *zhangwuensis* in the field avoid drought stress at noon, thus avoiding photoinhibition. Contrarily, *P. sylvestris* var. *mongolica* is a shallow-rooted tree species, and its sun branches have low potential hydraulic transport capacity, which is also partly the reason for its photoinhibition.

### 3.3. Heterogeneity of Photoinhibition in Different Needle Types

The position of needles affects the external environment, internal photosynthetic system formation, and photosynthesis of needles [35]. Unlike the sun CYNs, the fluorescence parameters such as F_v_/F_m_ of the shade CYNs of the two species exhibited relatively stable daily variations, and there was no photoinhibition phenomenon, which was related to the position of the shade CYNs that were shaded and exposed to weak light. A low photoinhibition of shade leaves has also been reported in other tree species [36]. Both species in our study maintained a high RC/CS_m_ under low light intensity, which is consistent with the reports of crops such as corn [37]. Although the RC/CS_m_ of shade CYNs of *P. densiflora* var. *zhangwuensis* could return to its morning level in the afternoon, this index decreased significantly after 16:00 in the shade CYNs of *P. sylvestris* var. *mongolica*, which may be related to the insufficient synthesis of key proteins caused by insufficient net photosynthetic products; thus, it needs a longer recovery time. Most of the time, the F_v_/F_m_ and F_m_/F_o_ values of the shade CYNs of the two species were higher than those of the sun CYNs, and the NPQ value was lower, indicating that they all reduced non-photochemical quenching under weak light conditions to transmit more light energy to the photoreaction center, and the electron transfer efficiency was improved, thereby ensuring a higher photochemical quantum yield of the chloroplast. The NPQ value of *P. densiflora* var. *zhangwuensis* was lower at noon, and the non-photochemical quenching pathway was almost completely inactive. Although the role of shade leaves in tree growth is rarely evaluated, their daily variations are relatively stable. A more consistent performance of the shade CYNs of the two species also shows that the main factor causing the difference in growth between the two species should not be present in the shade CYNs, but in the sun CYNs. Increasing the proportion of sun CYNs by pruning and other methods may be an effective way to increase the growth of *P. densiflora* var. *zhangwuensis*, which requires further experimental verification.

This study has some limitations. Due to the difficulty in investigating tall trees, the number of samples surveyed is not large. In the future, the number of survey samples will be increased to more strongly support some of the viewpoints presented by this study.

## 4. Materials and Methods

### 4.1. Study Area and the Experimental Forest

This study was carried out in the experimental forest of same-age *P. densiflora* var. *zhangwuensis* and *P. sylvestris* var. *mongolica* with consistent management measures in Zhanggutai (42° 43′ N, 122° 29′ E, altitude 226.5 m a.s.l.), Zhangwu County, Liaoning Province, China. The mean annual temperature, mean annual precipitation, mean pan evaporation, and ratio of evaporation to precipitation were 6.7 °C, 433 mm, 1570 mm, and 3.43–6.43, respectively. The soil was mainly sandy soil with a small amount of chestnut, saline–alkali, and peat soil. The annual variation range of soil moisture content in the experimental forest was 1.5–9.5%, and the soil nutrients were generally poor. The organic matter content of 0~100 cm soil layer was 0.646%, total nitrogen and total phosphorus were 0.017% and 0.019%, and pH was 6.27. The experimental period of this study was from 1 April 2020, to 11 June 2022. In order to avoid the interference of drought and nutrient deficiency on the study results of this study, the water management of the experimental forest was carried out to ensure that the soil water content was maintained at 60% field capacity. In June and July 2020 and 2021, the experimental forest was fertilized once each month, and the fertilizer type was compound fertilizer + urea (8:1) (5 kg·plant^−1^).

### 4.2. Determination of Experimental Forest Growth and Selection of Sample Branches

We randomly set up 6 standard plots (3 plots for each species) in the experimental forest, and the standard plot size was 30 m × 30 m. The growth of trees in each plot was investigated from November 3 to 5, 2020. The results showed that the height of *P. densiflora* var. *zhangwuensis* was between 6.66 and 8.39 m, and its average height was significantly higher than that of *P. sylvestris* var. *mongolica* of the same age, that is, about 1.11 times. The DBH of the former was between 9.37 cm and 23.89 cm, and its mean value was significantly higher than that of the latter, which was about 1.44 times. The coefficient of variation in the two growth indices of *P. densiflora* var. *zhangwuensis* is small, indicating the small growth dispersion of tree height and DBH, and the growth of individual trees tends to be more consistent (Table 4). Based on their average growth, 3 standard trees were selected for each species (one tree for one plot), and their trunks were marked with red self-spraying paint. On 1 June 2021, using the mobile lifting platform (Zhengzhou Yichu Machinery Equipment Co., Ltd., Zhengzhou, China), three current-year branches were randomly selected from the sunny part of the standard tree crown at about 6.5 m as sun branches, with another three current-year branches from the shady part of the crown at about 3.5 m as shade branches (Figure 6), and the growth of these sample branches was also measured. The results showed that the growth of sun branches of *P. densiflora* var. *zhangwuensis* was significantly higher than that of *P. sylvestris* var. *mongolica* and its own shade branches, while there was no significant difference between the two types of branches in *P. sylvestris* var. *mongolica* (Table 4).

### 4.3. Determination of Meteorological Factors and Fluorescence Parameters

The air temperature (Tair) and relative humidity (RH) data of the study period were obtained from a small weather station in the experimental forest. The light intensity around the selected branches of the two species was measured by a spectral color illuminometer (EVERFINE Corporation, Hangzhou, China) (Table 5). The results showed that the sun CYNs of the two species (growing on the sun-exposed side of the sun branches) could be directly exposed to sunlight for more than 8 h, and they were exposed to a high level of PAR in the range of 990–2054 μmol·m^−2^·s^−1^; the shade CYNs (growing on the shaded side of shade branches) were not exposed to direct sunlight throughout the day, and they were exposed to a low level of PAR in the range of 54–263 μmol·m^−2^·s^−1^.

On 8–10 June 2021, it was a continuously sunny day. Three bundles of sun CYNs were selected on each sun branch, and three bundles of shade CYNs were selected on each shade branch. The selected needles were marked with white cloth strips for further detection in the second year. The chlorophyll a fluorescence parameters of sun and shade CYNs of two species were determined using a Pocket PEA plant efficiency analyzer (Hansatech Instruments, London, UK) on the mobile lifting platform. The determination was carried out every 1 h from 8:00 to 17:00 for 3 days. The same needles were tracked and measured in June of the second year (9–11 June 2022), when the needles were 1-year-old needles (OYNs).

The determination of fluorescence parameters comprises two steps. In the first step, the initial fluorescence (F_o_), maximum fluorescence (F_m_), and variable fluorescence (F_v_) were measured after 20 min of dark adaptation for each needle; then, F_v_/F_m_ and F_m_/F_o_ were obtained. The second step measured the maximum steady-state fluorescence (F_m_′) after 15 min under natural light conditions. When saturated light (3500μmol·m^−2^·s^−1^) is used to measure F_m_′, the measurement must be performed within 1–2 s after the needles are clamped [38]. In the first step, the dark adaptation time of this study was determined by setting a series of dark adaptation time gradients; that is, the maximum light intensity of 3500 μmol·m^−2^·s^−1^ was selected to determine the F_v_/F_m_ value under different dark adaptation time gradients. When the F_v_/F_m_ value no longer increased with time, it was the best dark adaptation time (20 min in this study). The NPQ value was calculated according to Formula (1) [39]. The corresponding fluorescence values at 0.05 ms, 0.1 ms, 0.3 ms, 2 ms, and 30 ms can also be measured and marked as F_1_, F_2_, F_3_, F_4_, and F_5_, respectively. The values of ABS/RC and RC/CS_m_ can be calculated using the above fluorescence values according to Formulas (2) and (3).

(1) $$\mathrm{NPQ}=\frac{F_{m}}{F_{m}^{\prime }}-1$$

(2) $$\mathrm{ABS}/\mathrm{RC}=\frac{4\times \left(F_{3}-F_{o}\right)}{F_{4}-F_{o}}\times \frac{F_{m}}{F_{v}}$$

(3) $$\mathrm{RC}/{\mathrm{CS}}_{m}=\frac{0.25\times F_{v}}{F_{m}}\times \frac{F_{4}-F_{o}}{F_{3}-F_{o}}\times F_{o}$$

### 4.4. Determination of Morphological Indices of Sample Branch Tracheids

The sample branches of the two species were longitudinally cut into chips with a blade, and their thickness was about 2 mm. Then, the chips were immersed in water, and after the air was removed, they were placed in a mixture of 10% glacial acetic acid and 30% hydrogen peroxide (1:1 *v*/*v*) for separation. The separation solution was placed in a disposable plastic cup and placed in a digital thermostatic water bath (Jie Cheng Experimental Apparatus, Shanghai, China). The temperature was maintained at 65–70 °C until the chip sample softened and separated. The softened sample was washed with water and shaken in an ethanol solution so that a single tracheid could be separated and stained with safranin. Each sample was made into 2 slices, and each slice was observed and photographed with a computerized biological microscope (Shanghai Caikang Optical Instrument Co., Ltd., Shanghai, China) to form a bitmap-format photo. The length (mm), width (μm), and lumen diameter (μm) of tracheids, as well as the outer diameter (μm) and inner diameter (μm) of pits, were measured using the image analysis system 10.0 software (Beijing, China). A total of 20 tracheid indices were determined and averaged for each sample branch. Then, the mean value of the three sample branches was taken as the value of the different types of sample branches of each standard tree, and the significance test was carried out.

Theoretical hydraulic conductance (Kt, m^4^ MPa^−1^ s^−1^) was calculated according to the Hagen–Poiseuille equation [40]:

Kt = πρ D^4^/ 128 η (4)

where ρ denotes the density of water (5.55 × 10^7^ mmol m^−3^), η denotes the viscosity of water (1.002 × 10^−9^ MPa at 20 °C), and D denotes the lumen diameter (m).

### 4.5. Data Processing

The study data are reported as mean ± standard deviations except in special circumstances. The IBM SPSS Statistics (version 26; IBM, NY, USA) and Excel software (version 2019; Microsoft, WA, USA) were used to process and plot the data. Independent sample T test or one-way analysis of variance (ANOVA) was used to compare significant differences between the mean values of two or three groups of data (*p* < 0.05). Although the advantage of chlorophyll fluorescence technology is that it can perform non-destructive tracking surveys on the same bunch of needles, the measured data inevitably exhibit non-independence. Therefore, the repeated measurement analysis of variance method (Repeated Measurement ANOVA) was used to test the significance of data comparisons at different time points, and the Spearman correlation coefficient was used in correlation analysis.

## 5. Conclusions

*P. sylvestris* var. *mongolica* is the main cultivated tree species in the three northern regions of China. Although it exhibits photoinhibition under high light intensity, photoinhibition does not cause tree death, mainly because the tree species has mechanisms for adapting to photoinhibition. To adapt to the high light intensity environment in the sandy land, *P. sylvestris* var. *mongolica* utilizes two mechanisms, that is, heat dissipation and light energy transfer, both of which cause higher light energy loss. However, the healthy growth of *P. densiflora* var. *zhangwuensis* in the sandy area benefits from its adaptation to high light intensity, and its adaptation mechanisms are different from those of *P. sylvestris* var. *mongolica*. There was almost no photoinhibition observed in *P. densiflora* var. *zhangwuensis* under high light intensity because it exhibited a more stable F_v_/F_m_, higher RC/CS_m_ and F_m_/F_o_, a lower midday NPQ, and anatomical traits associated with a higher calculated Kt. These characteristics may contribute to the differential tolerance to high light intensity, thus avoiding photoinhibition and ensuring higher photosynthetic intensity. Regarding the correlations between the above conclusions and the decline in *P. sylvestris* var. *mongolica* forest and the selection of coniferous tree species in sandy land, it is necessary to conduct comparative experiments in multiple locations and years in future studies and to determine the D1 protein expression index to further prove these correlations.

## Figures and Tables

**Figure 1:**
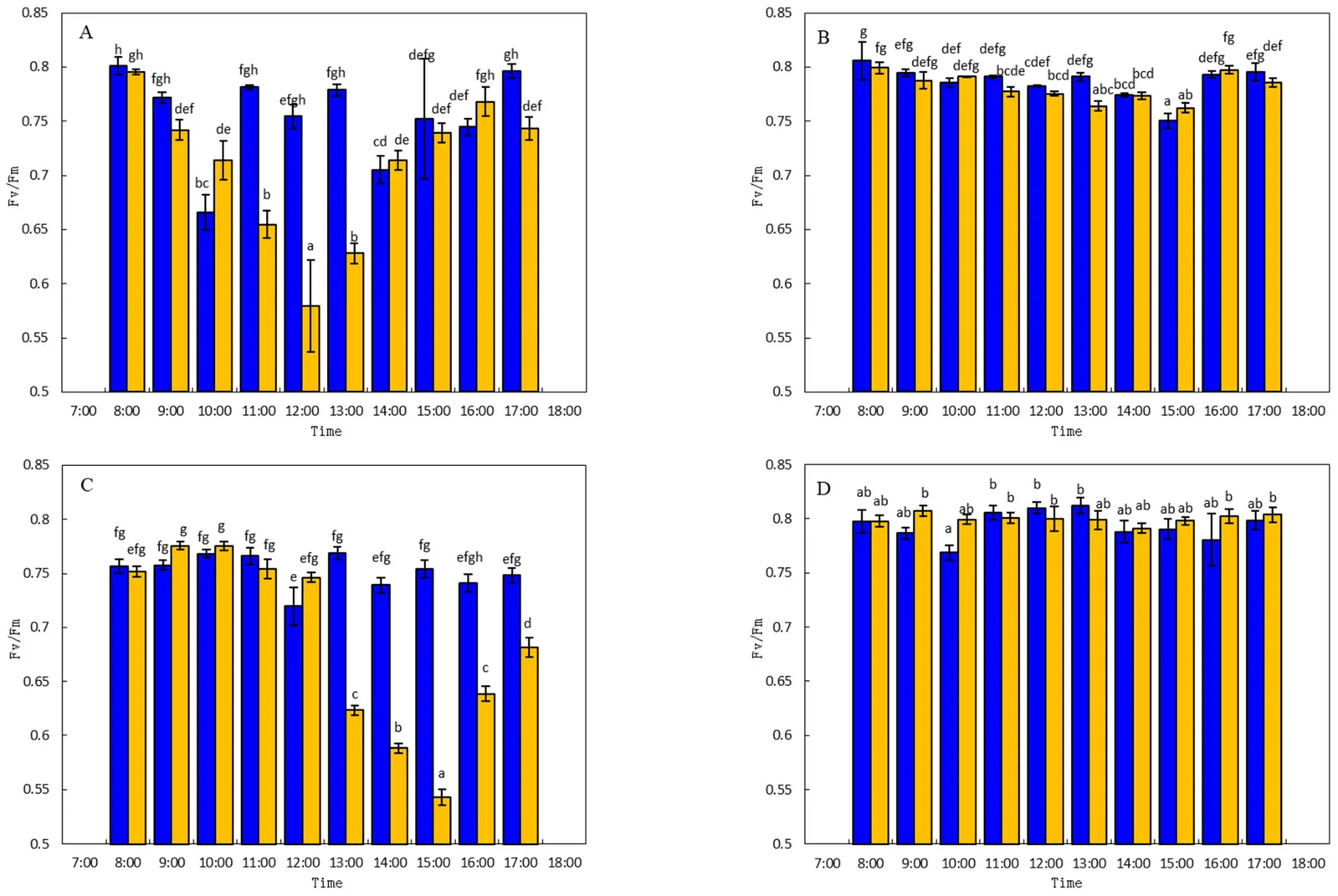
The daily changes in F_v_/F_m_ of sun CYNs (**A**), shade CYNs (**B**), sun OYNs (**C**), and shade OYNs (**D**) of *P. densiflora* var. *zhangwuensis* (blue column) and *P. sylvestris* var. *mongolica* (orange column). Error bars show SE calculated from three experimental repetitions (three trees). The same letter at the top of the column indicates that there is no significant difference in F_v_/F_m_ values under different treatments (*p* < 0.05).

**Figure 2:**
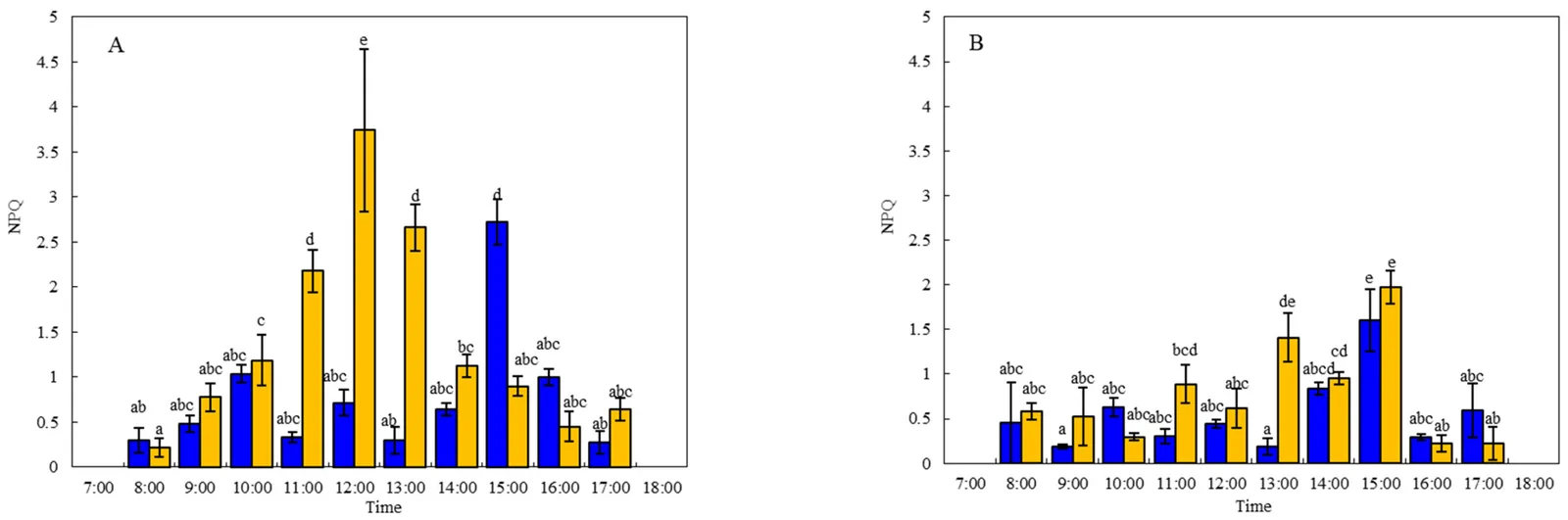
The daily changes in NPQ in the sun (**A**) and shade (**B**) CYNs of *P. densiflora* var. *zhangwuensis* (blue column) and *P. sylvestris* var. *mongolica* (orange column). Error bars show SE calculated from three experimental repetitions (three trees). The same letter at the top of the column indicates that there is no significant difference in NPQ values under different treatments (*p* < 0.05).

**Figure 3:**
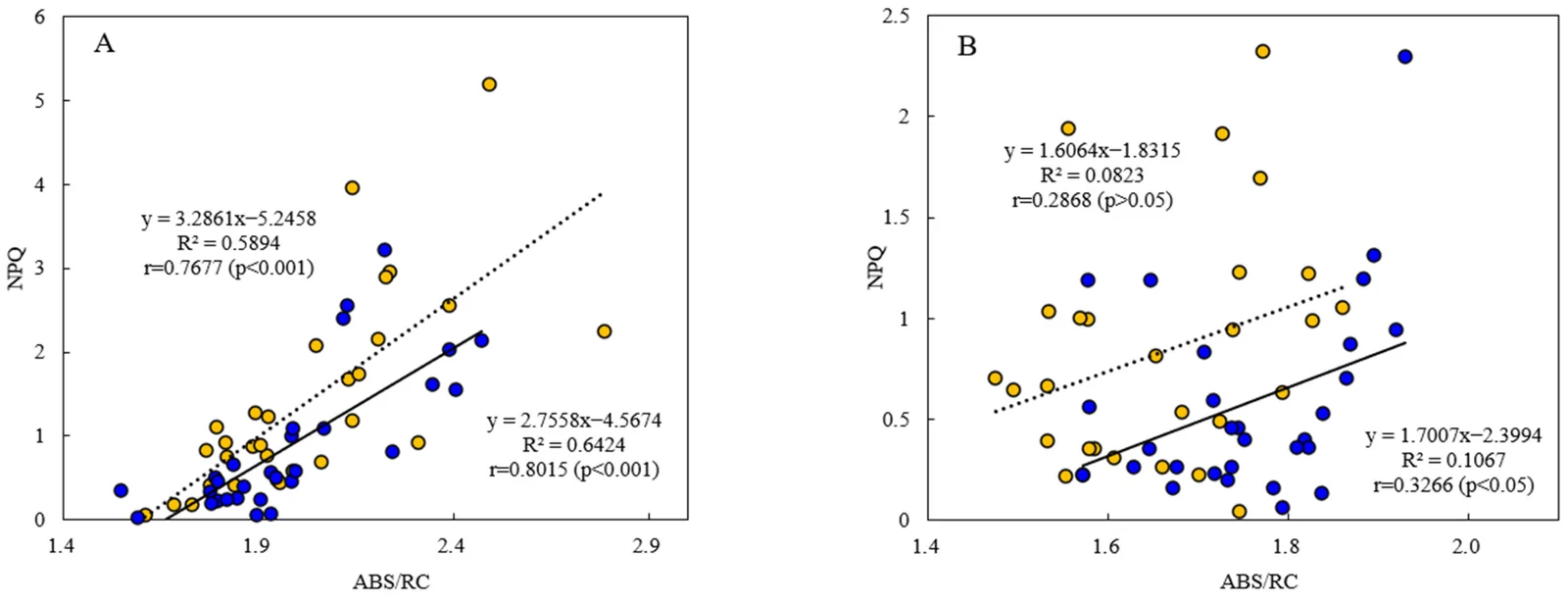
Correlation between NPQ and ABS/RC for the sun (**A**) and shade (**B**) needles of the two species. The blue and orange circles represent the data of *P. densiflora* var. *zhangwuensis* and *P. sylvestris* var. *mongolica*, respectively (*n* = 28–30). The solid and dotted trendlines represent the correlation between the two indices of *P. densiflora* var. *zhangwuensis* and *P. sylvestris* var. *mongolica*, respectively.

**Figure 4:**
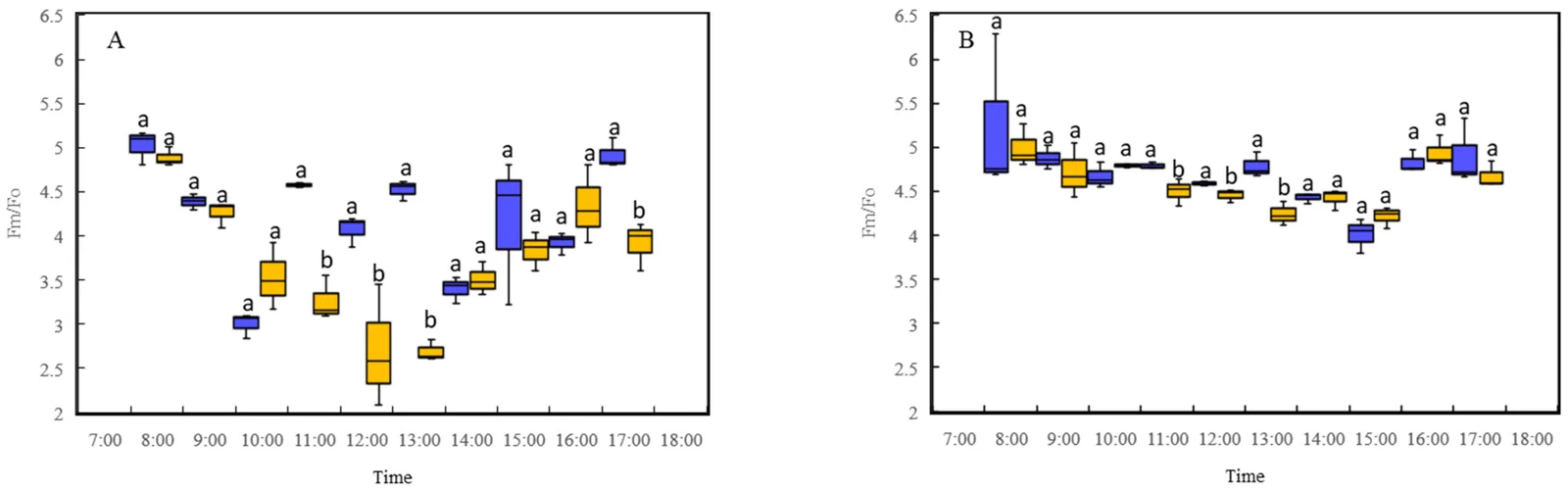
The daily changes in F_m_/F_o_ of the sun (**A**) and shade CYNs (**B**) of *P. densiflora* var. *zhangwuensis* (blue box) and *P. sylvestris* var. *mongolica* (orange box). The same letter at the top of the box indicates that there is no significant difference in F_m_/F_o_ values under different treatments (*p* < 0.05).

**Figure 5:**
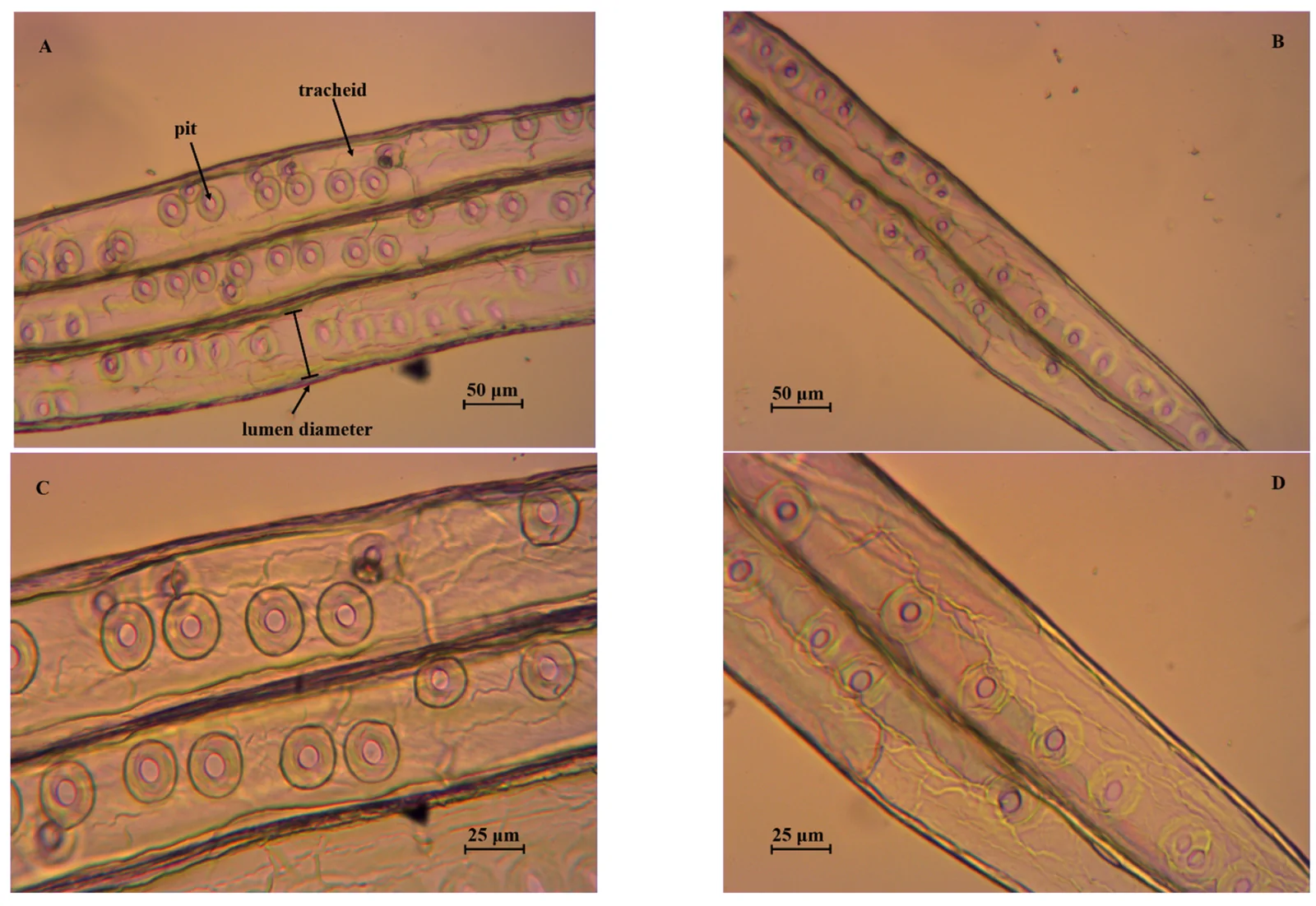
Morphological characteristics of the tracheids in the sun branches of *P. densiflora* var. *zhangwuensis* ((**A**) low-magnification; (**C**) high-magnification) and *P. sylvestris* var. *mongolica* ((**B**) low-magnification; (**D**) high-magnification).

**Figure 6:**
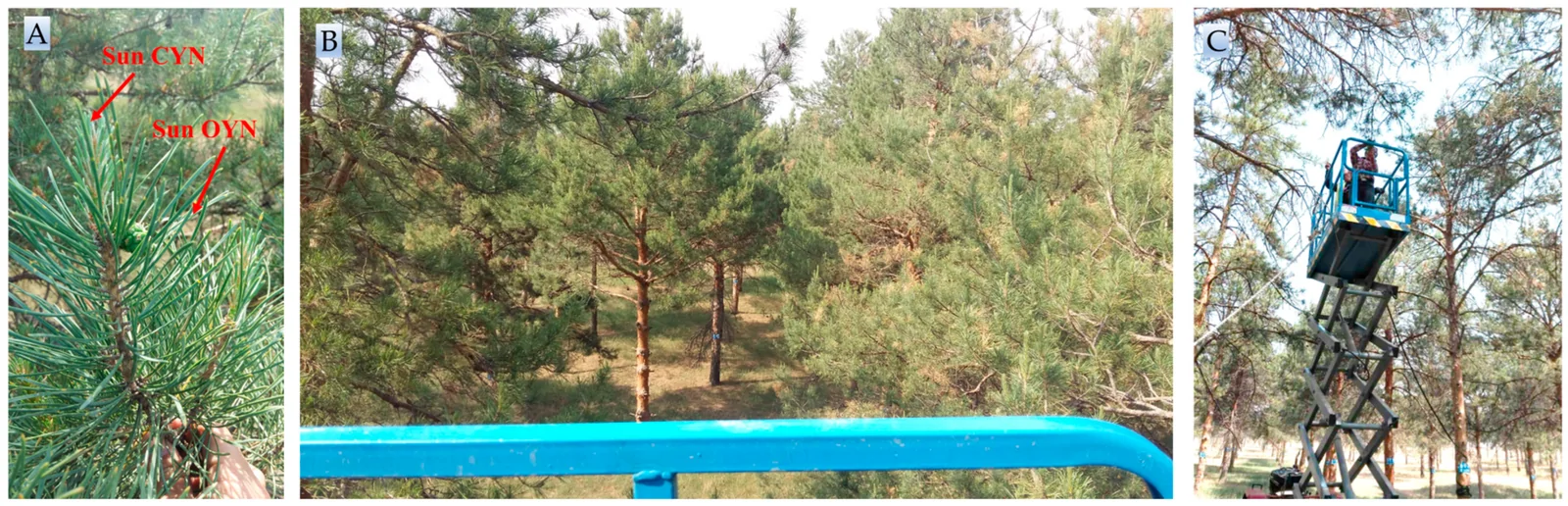
Sample branches (**A**) in the sunny part (**B**) of the crown of *P. sylvestris* var. *mongolica* selected using the mobile lifting platform (**C**). The red arrows in (**A**) indicate the positional relationship between sun CYNs and sun OYNs on the sample branch of *P. sylvestris* var. *mongolica*.

**Table 1:** The daily changes in ABS/RC and RC/CS_m_ of *P. densiflora* var. *zhangwuensis* and *P. sylvestris* var. *mongolica.*

Time	8:00	9:00	10:00	11:00	12:00
ABS/RC in sun CYNs of *P. densiflora* var. *zhangwuensis*	1.64 ± 0.07 Aa	1.87 ± 0.06 Aa	2.15 ± 0.03 Aa	1.85 ± 0.00 Aa	1.97 ± 0.02 Aa
ABS/RC in shade CYNs of *P. densiflora* var. *zhangwuensis*	1.54 ± 0.04 Aa	1.72 ± 0.03 Aa	1.72 ± 0.01 Ab	1.74 ± 0.00 Ab	1.80 ± 0.03 Ab
ABS/RC in sun CYNs of *P. sylvestris* var. *mongolica*	1.69 ± 0.05 Aa	2.02 ± 0.15 Aa	2.11 ± 0.03 Aa	2.44 ± 0.18 Ba	2.23 ± 0.13 Aa
ABS/RC in shade CYNs of *P. sylvestris* var. *mongolica*	1.50 ± 0.02 Ab	1.55 ± 0.01 Bb	1.58 ± 0.02 Bb	1.74 ± 0.01 Ab	1.77 ± 0.04 Ab
RC/CSm in sun CYNs of *P. densiflora* var. *zhangwuensis*	7859 ± 351 Aa	6450 ± 565 Aa	3697 ± 133 Aa	7613 ± 366 Aa	4720 ± 783 Aa
RC/CSm in shade CYNs of *P. densiflora* var. *zhangwuensis*	10398 ± 723 Ab	9416 ± 65 Ab	8415 ± 53 Ab	8515 ± 57 Aa	8298 ± 186 Ab
RC/CSm in sun CYNs of *P. sylvestris* var. *mongolica*	7540 ± 102 Aa	2006 ± 386 Ba	4066 ± 795 Aa	1679 ± 363 Ba	1575 ± 399 Ba
RC/CSm in shade CYNs of *P. sylvestris* var. *mongolica*	8620 ± 47 Ab	5690 ± 215 Bb	5466 ± 194 Ba	7118 ± 269 Bb	6262 ± 460 Bb
Time	13:00	14:00	15:00	16:00	17:00
ABS/RC in sun CYNs of *P. densiflora* var. *zhangwuensis*	1.79 ± 0.01 Aa	2.38 ± 0.02 Aa	2.06 ± 0.21 Aa	2.10 ± 0.07 Aa	1.93 ± 0.01 Aa
ABS/RC in shade CYNs of *P. densiflora* var. *zhangwuensis*	1.82 ± 0.01 Aa	1.88 ± 0.02 Ab	1.90 ± 0.01 Aa	1.65 ± 0.01 Ab	1.68 ± 0.07 Ab
ABS/RC in sun CYNs of *P. sylvestris* var. *mongolica*	2.22 ± 0.01 Ba	1.90 ± 0.01 Ba	1.79 ± 0.02 Aa	1.83 ± 0.06 Ba	1.95 ± 0.02 Aa
ABS/RC in shade CYNs of *P. sylvestris* var. *mongolica*	1.75 ± 0.10 Ab	1.59 ± 0.04 Bb	1.76 ± 0.01 Ba	1.66 ± 0.05 Aa	1.65 ± 0.04 Ab
RC/CSm in sun CYNs of *P. densiflora* var. *zhangwuensis*	8396 ± 279 Aa	4151 ± 214 Aa	4080 ± 1776 Aa	4218 ± 615 Aa	8409 ± 534 Aa
RC/CSm in shade CYNs of *P. densiflora* var. *zhangwuensis*	8414 ± 180 Aa	7986 ± 141 Ab	8125 ± 193 Aa	8897 ± 155 Ab	11637 ± 943 Ab
RC/CSm in sun CYNs of *P. sylvestris* var. *mongolica*	1399 ± 95 Ba	3221 ± 668 Aa	4615 ± 80 Aa	4345 ± 1072 Aa	5537 ± 224 Ba
RC/CSm in shade CYNs of *P. sylvestris* var. *mongolica*	6870 ± 314 Bb	7637 ± 68 Ab	7164 ± 39 Bb	7227 ± 348 Ba	3872 ± 365 Bb

Different lowercase letters indicate that the sun CYNs and shade CYNs of the same species are significantly different, and uppercase letters indicate that the two species with the same type of needles are significantly different (*p* < 0.05). The data in the table are presented as mean ± SE.

**Table 2:** General linear model univariate analysis of inter-subject effect (dependent variable: NPQ).

Sample Type	Source	Type III Sum of Squares	Degree of Freedom	Mean Square	F-Value	Significance
Sun needles	Adjusted model	36.064 ^a^	2	18.032	35.250	<0.001
	Intercept	21.099	1	21.099	41.245	<0.001
	Species	2.118	1	2.118	4.140	0.047
	ABS/RC	32.339	1	32.339	63.217	<0.001
Shade needles	Adjusted model	2.589 ^b^	2	1.295	4.978	0.010
	Intercept	0.973	1	0.973	3.739	0.058
	Species	1.938	1	1.938	7.449	0.009
	ABS/RC	1.742	1	1.742	6.695	0.012

^a^ R^2^ = 0.557 (Adjusted R^2^ = 0.541). ^b^ R^2^ = 0.156 (Adjusted R^2^ = 0.124).

**Table 3:** Comparison of tracheid characteristics of different branches between *P. densiflora* var. *zhangwuensis* and *P. sylvestris* var. *mongolica.*

Species	Branch Type	Tracheid Length (mm)	Tracheid Width (μm)	Lumen Diameter (μm)	Outer Diameter of Pit (μm)	Inner Diameter of Pit (μm)	**Kt (mm^4^ MPa^−1^ s^−1^)**
*P. densiflora* var. *zhangwuensis*	Sun	3.09 ± 0.44 Aa	36.56 ± 9.07 Aa	30.67 ± 6.25 Aa	14.95 ± 0.60 Aa	5.56 ± 0.33 Aa	1.20 × 10^−3^
	Shade	2.84 ± 0.58 Aa	37.86 ± 6.93 Aa	30.59 ± 10.90 Aa	15.06 ± 2.58 Aa	5.98 ± 1.37 Aa	1.19 × 10^−3^
*P. sylvestris* var. *mongolica*	Sun	2.00 ± 0.57 Ba	25.40 ± 7.05 Ba	19.40 ± 1.58 Ba	11.91 ± 0.86 Ba	4.35 ± 0.33 Ba	0.19 × 10^−3^
	Shade	2.50 ± 0.46 Bb	33.63 ± 7.24 Ab	28.07 ± 9.17 Ab	14.84 ± 1.92 Ab	5.72 ± 1.55 Ab	0.84 × 10^−3^

Different uppercase letters indicate significant differences between species in the same types of branches, and different lowercase letters indicate significant differences between different types of branches in the same species (*n* = 3, three trees for each treatment).

**Table 4:** Growth metrics of the two species and their sample branches.

Species	Tree Height (m)	DBH (cm)	Branch Type	Branch Length (cm)	Branch Thickness (mm)
*P. densiflora* var. *zhangwuensis*	7.59 ± 0.43 A	16.62 ± 2.54 A	Sun branch	43.4 ± 6.1 Aa	17.2 ± 2.0 Aa
	0.057 ^1^	0.153 ^1^	Shade branch	30.3 ± 5.0 Ab	11.7 ± 0.5 Ab
*P. sylvestris* var. *mongolica*	6.85 ± 0.82 B	11.54 ± 2.75 B	Sun branch	28.0 ± 0.5 Ba	11.6 ± 0.5 Ba
	0.120 ^1^	0.238 ^1^	Shade branch	26.8 ± 3.5 Aa	10.2 ± 1.1 Aa

Note: The age of the two species was 28 years when measured. The data with superscript 1 indicates the coefficient of variation. Different uppercase letters indicate that there are significant differences among tree species, and different lowercase letters indicate that there are significant differences between sun and shade branches of the same species (*p* < 0.05).

**Table 5:** The daily changes in some meteorological factors during the determination period of fluorescence indices in 2021 and 2022.

Factor	Time									
	8:00	9:00	10:00	11:00	12:00	13:00	14:00	15:00	16:00	17:00
Tair (°C) in 2021	17.7 ± 2.1 a	19.3 ± 1.5 a	21.7 ± 1.5 a	24.0 ± 2.0 a	25.7 ± 2.5 a	27.0 ± 2.0 a	27.3 ± 1.5 a	28.0 ± 1.0 a	28.0 ± 1.0 a	27.7 ± 0.6 a
Tair (°C) in 2022	18.1 ± 0.5 a	20.5 ± 0.2 a	22.1 ± 1.6 a	23.1 ± 1.4 a	26.9 ± 2.8 a	30.4 ± 0.4 b	29.2 ± 0.5 a	28.2 ± 0.4 a	29.3 ± 0.4 a	28.4 ± 0.1 a
RH (%) in 2021	58.5 ± 10.8 a	55.3 ± 14.0 a	51.0 ± 13.5 a	39.7 ± 9.5 a	28.3 ± 2.9 a	26.7 ± 2.9 a	21.0 ± 1.7 a	17.7 ± 4.2 a	17.0 ± 6.6 a	15.7 ± 5.1 a
RH (%) in 2022	55.8 ± 3.2 a	51.8 ± 0.5 a	46.1 ± 4.0 a	43.1 ± 2.1 a	24.8 ± 4.5 a	17.1 ± 0.6 b	23.2 ± 5.8 a	21.9 ± 0.5 a	23.6 ± 0.2 a	19.7 ± 1.5 a
Sun branch PAR (μmol·m^−2^·s^−1^)	990 ± 74 a	1672 ± 24 a	1795 ± 100 a	1928 ± 106 a	2054 ± 93 a	1948 ± 14 a	1932 ± 61 a	1380 ± 310 a	1128 ± 374 a	1113 ± 314 a
Shade branch PAR (μmol·m^−2^·s^−1^)	92 ± 1 b	110 ± 15 b	173 ± 3 b	210 ± 7 b	263 ± 3 b	185 ± 66 b	161 ± 32 b	137 ± 9 b	80 ± 1 b	54 ± 4 b

Note: There are no significant differences between the sun branches and shade branches of the two species. Therefore, the data in the above table only show the average values of the factors of the two species. Different lowercase letters indicate that the difference between the two years or between the sun branches and the shade branches is significant.

## Data Availability

The datasets generated and/or analyzed during the current study are available from the corresponding author on reasonable request.
